# The complete chloroplast genome sequence of *Actinidia styracifolia* C. F. Liang

**DOI:** 10.1080/23802359.2019.1698337

**Published:** 2019-12-11

**Authors:** Aihong Yang, Shujuan Liu, Tengyun Liu, Miao Hu, Yongda Zhong, Lipan Liu, Faxin Yu

**Affiliations:** The Key Laboratory of Horticultural Plant Genetic and Improvement of Jiangxi, Institute of Biological Resources, Jiangxi Academy of Sciences, Nanchang, Jiangxi, China

**Keywords:** *Actinidia styracifolia*, complete chloroplast genome, phylogenetic analysis

## Abstract

The complete chloroplast (cp) genome sequence of *Actinidia styracifolia* C. F. Liang was assembled using Illumina pair-end sequencing data in this study. The assembled plastome was 156,845 bp in length, including a large single copy (LSC) region of 88,624 bp and a small single copy (SSC) region of 20,535bp, which were separated by two inverted repeat (IR) regions of 23,843 bp. The plastome contains 113 different genes, consisting of 79 unique protein-coding genes, 30 tRNA genes, and 4 rRNA genes. Phylogenetic analysis based on chloroplast genomes revealed that *A. styracifolia* has a close genetic relationship with *A. eriantha.*

*Actinidia*, with 54 species and 21 varieties, is mainly distributed in East and South Asia (Huang et al. 2013). As an important fruit tree, *A. chinensis*, *A. chinensis* var. *deliciosa* and *A. arguta* have received considerable attention (Yao et al. 2015; Lin et al. 2018); however, little is known about narrowly distributed *Actinidia* species*. A. styracifolia* C. F. Liang is characterized by rich vitamin C in the fruits and now scattered distributed in Hunan, Guizhou, Jiangxi and Fujian province, China (Huang et al. 2013). It was recommended as Vulnerable species (VU) by the International Union for Conservation of Nature (IUCN). Knowledge of complete chloroplast (cp) genome would contribute greatly to reveal the phylogeny status and develop optimum conservation strategies for *A. styracifolia*. In this study, we assembled and characterized the complete chloroplast (cp) genome of *A. styracifolia* from Illumina pair-end sequencing data.

Fresh leaves were collected from a single individual of *A. styracifolia* sampled in Xunwu county (24°58′9″N, 115°47′44″E) of Jiangxi province, China. We preserved this sample into our Kiwifruit Germplasm Genebank (28°22′27″N, 116°0′59″E) by grafting. The voucher specimen (LBG00148294) was deposited in the Herbarium of Lushan Botanical Garden, Chinese Academy of Sciences (LBG). Genomic DNA was isolated using a modified CTAB method (Doyle and Doyle 1987). The whole-genome sequencing was obtained 150 bp paired-end reads using the Illumina Hiseq Platform. In total, 5.5 G raw reads were obtained, quality-trimmed and assembled against the plastome of *Actinidia chinensis* (GenBank: NC_026690.1) (Yao et al. 2015) using the program NOVOPlasty (Dierckxsens et al. 2017).

The complete chloroplast genome of *A. styracifolia* (GenBank accession number MN627226) exhibits the typical quadripartite structure of angiosperms, with a size of 156,845 bp in length. It comprises two inverted repeat (IR) regions of 23,843 bp separated by the large single-copy (LSC) region of 88,624 bp and small single-copy (SSC) region of 20,535 bp. The GC content of the LSC, SSC, and IRa/b regions is 35.45, 31.08 and 43.09%, respectively, and the overall GC content of the genome is 37.2%. The cp genome of *A. styracifolia* contains 113 unique genes, with 79 protein-coding genes, 30 tRNA genes, and 4 rRNA genes. The majority of the genes are single copy; however, 17 gene species in the IR regions are totally duplicated, including 5 protein-coding genes (*ycf2, ycf15, ndhB, rps7,* and *rps12*), 8 tRNA genes (*trnH-GUG, trnI-CAU, trnL-CAA, trnV-GAC, trnI-GAU, trnA-UGC, trnR-ACG*, and *trnN-GUU*) and all four rRNA genes. Compared with most other angiosperms, *A. styracifolia* lost its *clpP* gene, a conspicuous synapomorphic characteristic during the cp genome evolution of Actinidiaceae (Wang et al. 2016).

To confirm the phylogenetic position of *A. styracifolia*, eleven complete chloroplast genome sequences of Actinidiaceae were aligned using MAFFT v.7 (Katoh and Standley 2013), and maximum likelihood (ML) analysis was performed in RAxML8.0 (Stamatakis 2014) with 1000 bootstrap replicates. The phylogenetic analysis of 11 *Actinidia* chloroplast genomes showed that *A. styracifolia* was closely related to *A. eriantha* (Figure 1).

**Figure 1.**
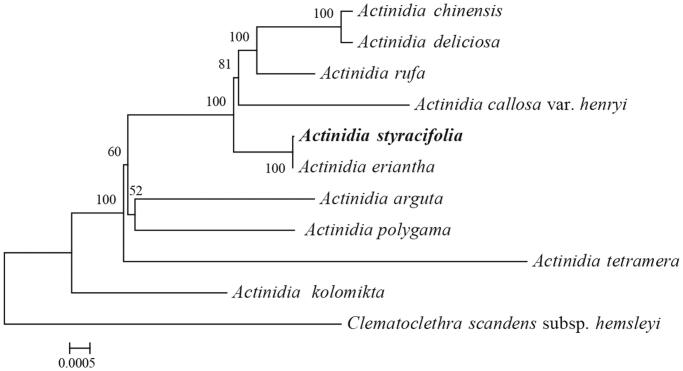
Phylogenetic relationships of 11 Actinidiaceae species based on complete chloroplast genomes. Bootstrap support values >50% are given at the nodes. GenBank accession numbers: *A. chinensis* (NC_026690.1), *A. deliciosa* (NC_026691.1), *A. rufa* (NC_039973.1), *A. callosa* var. *henryi* (NC_043861.1), *A. styracifolia* (MN627226), *A. eriantha* (NC_034914.1), *A. arguta* (NC_034913.1), *A. polygama* (NC_031186.1), *A. tetramera* (NC_031187.1), *A. kolomikta* (NC_034915.1), *Clematoclethra scandens* subsp. *Hemsleyi* (KX345299.1).
